# The Impact of cognitive remediation combined with mindfulness and social skills training on social functioning and neural plasticity in early psychosis: preliminary results from a randomized clinical multicentric trial in Italy

**DOI:** 10.1192/j.eurpsy.2024.120

**Published:** 2024-08-27

**Authors:** C. Perlini

**Affiliations:** ^1^Department of Neurosciences, Biomedicine and Movement Sciences, Section of Clinical Psychology, University of Verona; ^2^Clinical Psychology Service, Verona University Hospital, Verona, Italy

## Abstract

**Abstract:**

Affective and non-affective psychoses are characterized by deficits in neuro and social cognition, which strongly impact the patient’s psychosocial functioning and health and social system. Recent literature suggests that such deficits could benefit from the innovative combination of evidence-based interventions.

This lecture aims to describe an Italian multisite (Verona, Milano, Pavia), longitudinal randomized controlled trial funded by the Italian Ministry of Health investigating the impact of Cognitive Remediation (CR) alone or combined with other approaches (namely, Mindfulness and Social Skills Training (SST)) on clinical, neuropsychological, social and brain-related outcomes in patients with a DSM5 diagnosis of affective or non-affective psychosis.

In our study, patients underwent clinical and neuropsychological evaluation at baseline (T0), end of treatment (T1), and six months post-treatment (T2), which consisted of nearly four months of CR, CR+ Mindfulness, or CR + SST. The cognitive assessment included the Brief Assessment of Cognition for Schizophrenia (BAC-S) or Affective Disorders (BAC-A) and the Executive and Social Cognition Battery (ESCB), specifically designed to identify impairments in social cognition and executive functions in patients’ real life. Participants underwent a 3T multimodal MRI, including structural and functional sequences at T0 and T1. We also recruited healthy controls for comparative brain mapping at T0.

The present lecture will provide an overview of the research project, along with some preliminary findings on the effect of CR alone or combined with other interventions on clinical and social functioning and brain plasticity, with a focus on the degree of durability and generalization of CR effects to patients’ real life. The study’s outcomes have the potential to inform clinical and rehabilitative settings and tailor combined therapeutic interventions.

**Disclosure of Interest:**

C. Perlini Grant / Research support from: Italian Ministry of Health GR-2016-02361283

